# Nutritional status, yield components, quality, and antioxidant performance of some mango cultivars in response to effect of Sukkary and Zebda rootstocks

**DOI:** 10.7717/peerj.21896

**Published:** 2026-10-29

**Authors:** Mahmoud Abdel-Sattar, Laila Y. Mostafa, Abdullah Alebidi, Abdel-Moety Salama, Alhussein S. A. Hamad, Omar Abd Alhares Khalil, Walaa S. Yousry, Rashid S. Al-Obeed

**Affiliations:** 1 Department of Plant Production, College of Food and Agriculture Sciences, King Saud University, Riyadh, Saudi Arabia; 2 Tropical Fruit Research Department, Horticulture Research Institute, Agricultural Research Centre, Giza, Egypt; 3 Biotechnology of Natural Product, Technical University Munich, Freising, Germany

**Keywords:** Antioxidant compounds, Chemical characteristics, Mangifera indica, Nutrient contents, Physical characteristics, Sustainable, Tree growth

## Abstract

Rootstock selection critically influences mango scion performance. To achieve optimal productivity, this study evaluated growth, yield, fruit quality, and leaf nutrients of ‘Tommy Atkins’, ‘Osteen’, and ‘R2E2’ grafted onto ‘Sukkary’ and ‘Zebda’ rootstocks over two seasons (2024–2025). The experiment followed a two-factor design (two rootstocks × three scions) arranged in a randomized complete block design. Sukkary rootstock increased trunk diameter (up to 22.6 cm), leaf area (73.1 cm^2^), and yield (55.1 kg/tree) when combined with Tommy Atkins. However, Zebda rootstock resulted in higher leaf N (2.23%) and magnesium (Mg) (0.29%) across scions. Conversely, R2E2 grafted onto Zebda rootstock promoted greater tree height, whereas canopy volume was greater for R2E2 on Sukkary in 2024 and on Zebda in 2025. Leaf nutrient analysis revealed significantly higher phosphorus (P) and potassium (K) concentrations in scions grafted onto Zebda rootstock, while nitrogen (N) and magnesium (Mg) contents varied by scion–rootstock combination. In addition, R2E2 trees on Sukkary rootstock produced fruits with greater fruit weight, pulp weight, and larger fruit dimensions. Among the evaluated cultivars , Osteen demonstrated superior fruit quality compared to Tommy Atkins and R2E2, exhibiting higher levels of total soluble solids (TSS), titratable acidity (TA), total sugars, reducing sugars, vitamin C, and total carotenoid content. Overall, the results highlight the important role of rootstock in regulating scion vigor and fruit yield, with comparatively small effects on fruit quality characteristics. Based on these findings, grafting Tommy Atkins onto Sukkary is recommended for high-density planting, while grafting Osteen onto either rootstock optimizes fruit quality.

## Introduction

The family Anacardiaceae comprises mainly tropical and warm-subtropical species, with over 850 species classified into 73 genera (Sankaran et al., 2021). Mangifera is a genus within the Anacardiaceae family that includes 69 morphologically identified species and is divided into two subgenera: Mangifera and Limus (Litz, 2009). It is reported to contain 41 species in all, but almost all the edible cultivars of mango belong to the single species *Mangifera indica*, which has been cultivated for at least 4,000 years, resulting in over 1,200 identified cultivars that produce attractive fruits (Abdel-Sattar, Makhasha & Al-Obeed, 2024; Makhasha, Al-Obeed & Abdel-Sattar, 2024). Despite cultivation across a wide range of environments, all commercial mango producers face major problems, including high production costs, comparatively low yields, and irregular bearing (Ahmad et al., 2025). However, the expansion of mango production is greatly constrained by the low and insufficient availability of improved-quality, true-to-type, and certified planting material. Scion and rootstock selection are seen as offering some of the best prospects for improvement in sustainable mango production, which offers the prospect of increasing mango yield and productivity without increasing production costs (Shivran et al., 2023).

Commercial mango cultivars are grafted onto rootstock, which significantly influences their growth, yield, and physiological processes in the scion cultivar (Rizwanullah et al., 2023). The right rootstock is important for the partial or total direct influence on canopy architecture, nutritional uptake, flowering, and fruit production, with differences in fruit quality and tolerance to unfavourable climatic conditions (Rebolledo-Martínez et al., 2019; Roberto, Novello & Fazio, 2025). Rootstocks provide a root system that anchors the tree and acts as an absorbing organ for water and mineral nutrients from the soil. Likewise, allowing for reduced application of fertiliser reduces the risk of nutrient leaching and related toxicity while maintaining fruit quality and productivity (Ganeshamurthy, Rupa & Shivananda, 2018; Zang et al., 2024). Rootstocks play a primary role in determining orchard efficiency, which in turn affects the success or failure of commercial orchards (Dhurve et al., 2023).

The adoption of selected rootstocks in orchard management has recently expanded due to intensified fruit production systems and the significant effects of scion-rootstock interactions on gas exchange, fruit set, yield, tree vigor, and fruit quality (Bosa et al., 2014; Sarkhosh et al., 2021). Such effects are often attributed to rootstock–scion interactions that modulate hormonal balance, protein partitioning, carbohydrate partitioning, and water and nutrient acquisition and assimilation (Rasool et al., 2020; Domingues et al., 2021). Numerous studies have demonstrated the substantial impact of rootstocks on scion cultivars; understanding these relationships is essential for selecting optimal graft combinations to improve orchard uniformity, manage tree vigor, and ensure consistent high yields and fruit quality (Dayal et al., 2016; Jain et al., 2025; Roberto, Novello & Fazio, 2025). To achieve optimal productivity, particularly with improved varieties, selecting an appropriate graft combination is crucial for enhancing water potential, nutrient uptake, plant vigor, yield efficiency, and fruit quality (Tietel et al., 2020; Shivran et al., 2023).

The improved varieties of mango grown in Egypt include Osteen, Haden, Keitt, Kent, R2E2, Tommy Atkins, and various others (Elshahawy et al., 2022). Many improved mango varieties perform poorly due to unsuitable rootstocks. There is no conclusive research on elite rootstock types and their effects on the nutritional status, yield components, and quality performance of improved mango cultivars (Vittal et al., 2023). Therefore, it is an important step for farmers uncertain about which rootstocks to use in sustainable agricultural management. This approach offers a practical solution that may improve productivity without increasing input costs and can modify sensory properties to meet market requirements (Rebolledo-Martínez et al., 2019). In this context, the present study was conducted to evaluate tree growth, leaf tissue nutrient concentrations, yield components, and the physical, chemical, and antioxidant properties of ‘Tommy Atkins’, ‘Osteen’, and ‘R2E2’ mangoes grafted onto the polyembryonic rootstocks Sukkary and Zebda.

## Materials & Methods

### Experimental site and plant materials

This investigation was carried out during two consecutive seasons, 2024 and 2025, on mango trees (*Mangifera indica* L.) grown in Wadi El-Nouqra, Nasr Nuba Center, Aswan Governorate, Egypt (GPS coordinates: 24°25′17.4′′N, 33°14′53.7′′E). Three cultivars, ‘Tommy Atkins’, ‘Osteen’, and ‘R2E2’ on ‘Sukkary’ and ‘Zebda’ rootstocks, were used for the experiment. To obtain an apomictic root system as a clone of the Sukkary and Zebda rootstock mother trees, the seeds are sown in a culture medium until the seedlings reach 20 cm in height. At that point, only the germinated vegetative embryos are separated and transplanted singly into polyethylene bags and then grafted onto them. Sexual seedlings are discarded and not transplanted into nursery bags (Singh et al., 2025). Trees were 8 years old, planted at a five × four m space under drip irrigation with 8 emitters (4 L h^−1^) per tree, two lines/tree row running parallel. The soil was sandy clay loam with an average of 7.84 pH, 1.63 dS m^−1^ E.C., 4.84 meq/L Ca^2+^, 2.69 meq/L Mg^2+^, 8.19 meq/L Na^+^, 0.55 meq/L K^+^, 0.65 meq/L HCO_3_^−^, 12.93 meq/L Cl^−^, 0.27% Organic material, 3.63% CaCO_3_, 39.67 mg kg^−1^ N, 7.33 mg kg^−1^ P, and 178.66 mg kg^−1^ K across seasons. Mango orchards were managed according to conventional practices in the area, using the same fertilization, protection from diseases, insect control, and other cultural practices.

### Experimental design and treatments

The design of the experiment was completely randomized (RCBD) as a factorial experiment with two factors and four replications consisting of single-tree plots for each rootstock-scion combination. The first factor included two rootstocks, namely Sukkary and Zebda. The second factor included three scions, namely Tomy Atkins, Osteen, and R2E2. The experiment included 24 trees in total, with 12 trees grafted onto Sukkary and 12 onto Zebda rootstock.

### Measurement and determinations

The nutritional status of mango trees was assessed by determining tree growth and leaf tissue nutrient contents. Tree growth was assessed by determining tree height, leaf area, trunk diameter, and canopy volume. The leaf area measurement was determined on the complete leaf using a digital planimeter. Tree height (H) was measured using a clinometer instrument. A measuring tape was used to determine tree spreading (m) and trunk circumference (TC) (two cm above the graft point). To determine the spread, it was measured twice on both the east and west sides and the north-south sides. Tree spread [North-South (D1)] and [E-W (Dr)] was recorded one month before flowering in February with the help of a meter scale. Canopy volume was calculated according to Zekri (2000). While alternative models exist, this formula is widely used in mango and provides consistent relative rankings among treatments.

(1) \begin{eqnarray*}\text{Canopy volume}~({\mathrm{m}}^{3})=(\pi /6)\times \mathrm{H}\times \mathrm{D1}\times \mathrm{Dr}\end{eqnarray*}

Trunk diameter was calculated by the formula:

(2) \begin{eqnarray*}\text{Trunk diameter}~(\mathrm{m})=\mathrm{TC}/\pi .\end{eqnarray*}

For the determination of nutrient status, twenty mature leaves were collected from non-fruiting terminal shoots (approximately 5–7 months old, healthy, and the 4th to 6th leaf from the apex) according to Bhargava & Chadha (1988). The leaves were oven-dried at 65 °C until a constant mass. They were then ground and digested with sulphuric acid and hydrogen peroxide according to Evenhuis & De Waard (1980). Total nitrogen was determined by the Micro–Kjeldahl method as described by Barbano et al. (1990). Phosphorus content was determined colorimetrically using a spectrophotometer at a wavelength of 882 nm according to the method described by Murphy & Riley (1962). Potassium content was determined using the flame photometric method (Jackson, 1958). The levels of magnesium (Mg) and calcium (Ca) were determined using an atomic absorption spectrophotometer, “Perkin Elmer 3300”, according to Jones (1977).

At maturity, the total fruit number per tree was recorded, and the harvested fruits were weighed separately for each replicate using an electronic scale. Tree fruit yield (kg) was estimated by the number per tree and the average fruit weight. Yield efficiency was determined by dividing the fruit yield per tree (Kg/tree) by the total canopy volume (m^3^).

At harvest, five fully mature fruits were sampled from each tree to evaluate physical characteristics. The average fruit weight (g) was estimated by weighing samples from each replicate. Fruit length and width were measured by a digital caliper (mm). Fruit shape, which was calculated by dividing fruit length/fruit width. The fruits were peeled, with the pulp, seed, and peel separated. Each fraction was weighed. Pulp weight (%) was calculated as: (pulp weight/fruit weight) × 100. Peel weight (%) was counted as: (peel weight/fruit weight) × 100. Seed weight (%) was counted as: (seed weight/fruit weight) × 100.

The total soluble solids (TSS) were determined using a hand refractometer (ATAGO, model N-20; Bellevue, WA, USA). Juice samples were filtered to estimate titratable acidity by the titration method against NAOH (0.1 N) with phenolphthalein as an indicator, and expressed in % citric acid according to standard methods (AOAC, 2019). Moreover, the TSS/titratable acidity ratio was also recorded. Total sugars percentage was measured by using the phenol sulfuric acid method according to Malik & Singh (1980). Fruit-reducing sugars content was measured colorimetrically according to the Lane and Eynon method as described by Egan, Kirk & Sawyer (1981).

Vitamin C content was assessed by titrating 5ml of filtered juice with 2% oxalic acid using 2,6-dichlorophenolindophenol dye to the end point according to the AOAC (2019) and expressed as milligrams of ascorbic acid/100 mL of juice. One gram of fruit pulp was extracted with 10 mL of acetone (85%), and carotenoid content was measured colourimetrically at 440 nm using a spectrophotometer, as described by Moran & Porath (1980). The amount of carotenoids was expressed as mg/100 g fruit weight (f.w.). Total phenolic content was determined following the procedure described by Swain & Hillis (1959), and the values were expressed as a percentage of the fresh weight of mango pulp.

### Statistical analysis

The experiment was carried out as a factorial in randomized complete block design RCBD in four replications. The studied factors were rootstock, scion, and their interaction on nutritional status, yield components, and fruit quality. All treatments were compared using two-factor ANOVA at the 0.95 confidence level. When they were significant (*p* < 0.05), a Least Significant Difference (LSD) test was used to compare means for significant differences using SAS 9.0 statistical software (SAS Statistical Package, 2008).

## Results

### Tree growth

The tree growth of different mango varieties, expressed in terms of trunk diameter, tree height, canopy volume, and leaf area, was significantly affected by rootstock, scion cultivar, and their interaction (Table 1). Regardless of rootstock, the Sukkary cultivar recorded the greatest trunk diameter and leaf area. In contrast, Zebda exhibited the highest values for tree height in both seasons and canopy volume in 2025 season. Among the scion cultivars, Tommy Atkins produced the highest trunk diameter and leaf area, followed by Osteen, whereas R2E2 recorded the lowest values for these parameters. Conversely, the R2E2 cultivar significantly enhanced tree height and canopy volume during both seasons. For canopy volume, the interaction between rootstock and scion was significant. In 2024, R2E2/Sukkary produced the largest canopy volume (39.77 m^3^), while in 2025, R2E2/Zebda produced the largest (41.76 m^3^). The main effect of rootstock on canopy volume was not significant in either season.

**Table 1:** Influence of rootstock, scion, and their interaction on tree growth of Tommy Atkins Osteen, and R2E2 mango cultivars grafted on Sukkary and Zebda rootstocks during the 2024 and 2025 seasons. Mean values representing different small letters, capital letters in columns and rows are significant between columns and rows, respectively, according to LSD (*p* < 0.05).

	**Season 2024**				**Season 2025**			
**Rootstock** **/** ** Scion**	**Tommy Atkins**	**Osteen**	**R2E2**	**Mean**	**Tommy Atkins**	**Osteen**	**R2E2**	**Mean**
	Trunk diameter (cm)							
**Sukkary**	22.62	20.00	18.52	20.38A	23.24	20.81	19.52	21.19A
**Zebda**	21.05	19.38	17.95	19.46B	21.80	19.24	18.36	19.80B
**Mean**	21.84a	19.69b	18.23c		22.52a	20.03b	18.94c	
**LSD at**_**0.05**_	Rootstock (RT)	Variety (V)		RT×V	Rootstock (RT)	Variety (V)		RT×V
	= 0.40	0.49		0.70	= 0.13	0.16		0.22
	Tree height (m)							
**Sukkary**	2.08	2.30	2.55	2.31B	2.13	2.35	2.54	2.34B
**Zebda**	2.20	2.38	2.49	2.35A	2.25	2.41	2.60	2.42A
**Mean**	2.14c	2.34b	2.52a		2.19c	2.38b	2.57a	
**LSD at**_**0.05**_	Rootstock (RT)	Variety (V)		RT×V	Rootstock (RT)	Variety (V)		RT×V
	= 0.04	0.05		0.07	= 0.03	0.04		0.06
	Canopy volume (m^3^)							
**Sukkary**	11.95	19.75	39.77	23.83A	12.86	21.03	34.89	22.93B
**Zebda**	15.07	22.81	33.14	23.67A	16.14	24.04	41.76	27.31A
**Mean**	13.51c	21.28b	36.46a		14.50c	22.54b	38.33a	
**LSD at**_**0.05**_	Rootstock (RT)	Variety (V)		RT×V	Rootstock (RT)	Variety (V)		RT×V
	= 1.69	2.07		2.92	= 1.03	1.26		1.78
	Leaf area (cm^2^)							
**Sukkary**	73.13	63.27	44.36	60.25A	74.80	65.93	50.83	63.85A
**Zebda**	68.89	60.91	40.13	56.64B	69.38	61.15	45.15	58.56B
**Mean**	71.01a	62.09b	42.24c		72.09a	63.54b	47.99c	
**LSD at**_**0.05**_	Rootstock (RT)	Variety (V)		RT×V	Rootstock (RT)	Variety (V)		RT×V
	= 0.79	0.97		1.37	= 0.37	0.45		0.64

### Leaf tissue nutrient contents

Leaf nutrient content was significantly influenced by both rootstock and scion cultivars. The mean effect of rootstocks indicated that Zebda rootstock enhanced phosphorus (P) and potassium (K) accumulation in both seasons compared to Sukkary. For example, in 2024, leaf P was 0.17% for Zebda *vs.* 0.16% for Sukkary; leaf K was 0.97% for Zebda *vs.* 0.92% for Sukkary. With respect to scion cultivar effects, R2E2 showed superiority in leaf N, P, K, Ca, and Mg contents across both seasons, followed by Osteen, while Tommy Atkins exhibited comparatively lower nutrient accumulation. The interaction between rootstock and scion significantly affected leaf nutrient status (N, P, K, Ca, and Mg) (Table 2). The R2E2/Zebda combination demonstrated the highest accumulation of all measured nutrients in both seasons, followed by the Osteen/Zebda combination. In contrast, Tommy Atkins grafted onto Zebda rootstock consistently exhibited the lowest leaf nutrient concentrations.

**Table 2:** Influence of rootstock, scion, and their interaction on leaf tissue nutrient contents of Tommy Atkins Osteen, and R2E2 mango cultivars grafted on Sukkary and Zebda rootstocks during the 2024 and 2025 seasons. Mean values representing different small letters, capital letters in columns and rows are significant between columns and rows, respectively, according to LSD (*p* < 0.05).

	**Season 2024**				**Season 2025**			
**Rootstock** **/** **Scion**	**Tomy Atkins**	**Osteen**	**R2E2**	**Mean**	**Tomy Atkins**	**Osteen**	**R2E2**	**Mean**
	Nitrogen (%)							
**Sukkary**	1.76	2.07	2.29	2.04A	1.88	2.15	2.43	2.15B
**Zebda**	1.83	2.13	2.36	2.11A	1.94	2.24	2.52	2.23A
**Mean**	1.80c	2.10b	2.32a		1.91c	2.19b	2.48a	
**LSD at**_**0.05**_	Rootstock (RT)	Variety (V)		RT×V	Rootstock (RT)	Variety (V)		RT×V
	= 0.08	0.10		0.14	= 0.06	0.08		0.11
	Phosphorus (%)							
**Sukkary**	0.15	0.18	0.16	0.16A	0.16	0.18	0.16	0.16A
**Zebda**	0.16	0.17	0.19	0.17B	0.18	0.19	0.20	0.19B
**Mean**	0.16b	0.17a	0.17a		0.17b	0.18a	0.18a	
**LSD at**_**0.05**_	Rootstock (RT)	Variety (V)		RT×V	Rootstock (RT)	Variety (V)		RT×V
	= 0.003	0.003		0.005	= 0.003	0.004		0.006
	Potassium (%)							
**Sukkary**	0.86	0.98	0.94	0.92A	0.88	1.05	0.97	0.97A
**Zebda**	0.88	1.01	1.02	0.97B	0.98	1.05	1.14	1.06B
**Mean**	0.87b	0.99a	0.98a		0.93b	1.05a	1.05a	
**LSD at**_**0.05**_	Rootstock (RT)	Variety (V)		RT×V	Rootstock (RT)	Variety (V)		RT×V
	= 0.015	0.018		0.025	= 0.015	0.018		0.025
	Calcium (%)							
**Sukkary**	1.85	1.96	1.90	1.90A	1.89	1.99	1.94	1.94B
**Zebda**	1.86	2.01	2.07	1.98B	1.89	1.98	2.14	2.00A
**Mean**	1.85b	1.98a	1.98a		1.89c	1.99b	2.04a	
**LSD at**_**0.05**_	Rootstock (RT)	Variety (V)		RT×V	Rootstock (RT)	Variety (V)		RT×V
	= 0.014	0.017		0.024	= 0.013	0.016		0.023
	Magnesium (%)							
**Sukkary**	0.27	0.28	0.28	0.28B	0.27	0.29	0.29	0.28B
**Zebda**	0.27	0.30	0.30	0.29A	0.27	0.29	0.31	0.29A
**Mean**	0.27b	0.29a	0.29a	0.30	0.270b	0.290a	0.296a	
**LSD at**_**0.05**_	Rootstock (RT)	Variety (V)		RT×V	Rootstock (RT)	Variety (V)		RT×V
	= 0.006	0.007		0.010	= 0.007	0.009		0.012

### Yield components

The yield components of the evaluated mango cultivars, expressed as fruit number per tree, total yield, and yield efficiency, were significantly affected by rootstock, scion cultivar, and their interaction (Table 3). The mean effect of rootstock indicated that Sukkary produced the highest fruit number, yield, and yield efficiency in both seasons, whereas the opposite response was observed when Zebda was used as a rootstock. Irrespective of rootstock, Tommy Atkins recorded the highest fruit number, total yield, and yield efficiency, followed by Osteen. In contrast, R2E2 exhibited the lowest yield component values, although these differences were not statistically significant compared with Osteen during the 2024 season. The interaction between rootstock and scion cultivar significantly influenced yield components; notably, Tommy Atkins grafted onto Sukkary rootstock produced the greatest fruit number, total yield, and yield efficiency in both seasons.

**Table 3:** Influence of rootstock, scion, and their interaction on yield components of Tommy Atkins Osteen, and R2E2 mango cultivars grafted on Sukkary and Zebda rootstocks during the 2024 and 2025 seasons. Mean values representing different small letters, capital letters in columns and rows are significant between columns and rows, respectively, according to LSD (*p* < 0.05).

	**Season 2024**				**Season 2025**			
**Rootstock** **/** **Scion**	**Tomy Atkins**	**Osteen**	**R2E2**	**Mean**	**Tomy Atkins**	**Osteen**	**R2E2**	**Mean**
	Fruit number							
**Sukkary**	181.75	114.00	79.00	124.92A	186.25	111.50	76.75	124.83A
**Zebda**	162.25	101.75	63.75	109.25B	165.00	99.75	62.00	108.92B
**Mean**	172.00a	107.88c	71.38c		175.63a	105.63b	69.38c	
**LSD at**_**0.05**_	Rootstock (RT)	Variety (V)		RT×V	Rootstock (RT)	Variety (V)		RT×V
	= 2.33	2.86		4.04	= 1.32	1.62		2.28
	Yield (kg/tree)							
**Sukkary**	63.55	54.35	47.42	55.11A	65.68	53.39	46.11	55.06A
**Zebda**	54.10	45.69	37.01	45.60B	55.65	45.04	36.12	45.61B
**Mean**	58.82a	50.02b	42.21c		60.67a	49.22b	41.11c	
**LSD at**_**0.05**_	Rootstock (RT)	Variety (V)		RT×V	Rootstock (RT)	Variety (V)		RT×V
	= 1.12	1.37		1.94	= 0.94	1.15 1.63	
	Yield efficiency							
**Sukkary**	4.85	2.50	1.08	2.81A	5.11	2.54	1.32	2.99A
**Zebda**	3.28	1.84	1.01	2.04B	3.46	1.88	0.87	2.07B
**Mean**	4.07a	2.17b	1.05c		4.28a	2.21b	1.09c	
**LSD at**_**0.05**_	Rootstock (RT)	Variety (V)		RT×V	Rootstock (RT)	Variety (V)		RT×V
	= 0.17	0.21		0.29	= 0.11	0.14		0.19

### Fruit physical characteristics

Fruit physical characteristics were significantly influenced by both rootstock and scion cultivar (Tables 4 and 5). With respect to rootstock effects, Sukkary recorded the highest values for most fruit physical traits, except for fruit peel weight, seed weight, and shape index. Zebda rootstock exhibited comparable performance to Sukkary for fruit peel weight and shape index during the first season, while in the second season, Zebda recorded the highest values for these traits. Regarding scion cultivar effects, R2E2 showed superior performance in most fruit physical characteristics across both seasons. Osteen and R2E2 displayed similar performance with respect to fruit shape index during the first season. The interaction between rootstock and scion cultivar significantly affected fruit physical characteristics of the studied mango cultivars grown on Sukkary and Zebda rootstocks (Tables 4 and 5). The R2E2/Sukkary combination exhibited superiority in fruit weight, pulp weight, and fruit length and width. However, during the first season, the R2E2/Sukkary combination exhibited superior fruit weight, pulp weight, and fruit dimensions compared to all other combinations in both seasons. In the second season, the R2E2/Zebda combination resulted in a significantly higher fruit shape index.

**Table 4:** Influence of rootstock, scion, and their interaction on fruit physical characteristics of Tommy Atkins Osteen, and R2E2 mango cultivars grafted on Sukkary and Zebda rootstocks during 2024 to 20252 seasons. Mean values representing different small letters, capital letters in columns and rows are significant between columns and rows, respectively, according to LSD (*p* < 0.05).

	**Season 2024**				**Season 2025**			
**Rootstock** **/** **Scion**	**Tomy Atkins**	**Osteen**	**R2E2**	**Mean**	**Tomy Atkins**	**Osteen**	**R2E2**	**Mean**
	Fruit weight (gm)							
**Sukkary**	349.80	476.68	600.16	475.55A	352.65	478.88	600.73	477.42A
**Zebda**	333.43	449.08	580.50	454.33B	337.28	451.58	582.53	457.13B
**Mean**	341.61c	462.88b	590.33a		344.96c	465.23b	591.63a	
**LSD at**_**0.05**_	Rootstock (RT)	Variety (V)		RT×V	Rootstock (RT)	Variety (V)		RT×V
	= 7.53	9.22		13.03	= 5.94	7.28		10.29
	Peel %							
**Sukkary**	5.66	5.44	5.07	5.39A	5.72	5.46	5.11	5.43B
**Zebda**	5.83	5.30	5.16	5.43A	5.88	5.34	5.19	5.47A
**Mean**	5.74a	5.37b	5.12c		5.80a	5.40b	5.15c	
**LSD at**_**0.05**_	Rootstock (RT)	Variety (V)		RT×V	Rootstock (RT)	Variety (V)		RT×V
	= 0.05	0.06		0.08	= 0.04	0.04		0.06
	Seed %							
**Sukkary**	17.45	12.80	12.18	14.14A	17.86	12.90	12.26	14.34B
**Zebda**	18.83	13.40	12.55	14.92B	18.91	13.59	12.77	15.09A
**Mean**	18.14a	13.10b	12.36c		18.38a	13.25b	12.51c	
**LSD at**_**0.05**_	Rootstock (RT)	Variety (V)		RT×V	Rootstock (RT)	Variety (V)		RT×V
	= 0.30	0.37		0.52	= 0.16	0.19		0.27
	Pulp %							
**Sukkary**	76.90	81.77	82.75	80.47A	76.42	81.64	82.63	80.23A
**Zebda**	75.35	81.30	82.30	79.65B	75.21	81.07	82.05	79.44B
**Mean**	76.12c	81.53b	82.53a		75.82c	81.36b	82.34a	
**LSD at**_**0.05**_	Rootstock (RT)	Variety (V)		RT×V	Rootstock (RT)	Variety (V)		RT×V
	= 0.28	0.35		0.49	= 0.14	0.18		0.25

**Table 5:** Influence of rootstock, scion, and their interaction on fruit shape characteristics of Tommy Atkins Osteen, and R2E2 mango cultivars grafted on Sukkary and Zebda rootstocks during 2024 to 20252 seasons. Mean values representing different small letters, capital letters in columns and rows are significant between columns and rows, respectively, according to LSD (*p* < 0.05).

	**Season 2024**				**Season 2025**			
**Rootstock** **/** **Scion**	**Tomy Atkins**	**Osteen**	**R2E2**	**Mean**	**Tomy Atkins**	**Osteen**	**R2E2**	**Mean**
	Fruit length (cm)							
**Sukkary**	13.40	15.95	16.85	15.40A	13.85	15.78	16.78	15.47A
**Zebda**	12.93	15.40	16.20	14.84B	12.98	15.58	16.35	14.97B
**Mean**	13.16c	15.68b	16.53a		13.42c	15.68b	16.56a	
**LSD at**_**0.05**_	Rootstock (RT)	Variety (V)		RT×V	Rootstock (RT)	Variety (V)		RT×V
	= 0.07	0.09		0.12	= 0.10	0.13		0.18
	Fruit width (cm)							
**Sukkary**	8.80	10.68	10.90	10.13A	9.30	10.58	11.00	10.29A
**Zebda**	8.58	9.95	10.68	9.73B	8.90	10.13	10.13	9.72B
**Mean**	8.69c	10.31b	10.79a		9.10c	10.35b	10.56a	
**LSD at**_**0.05**_	Rootstock (RT)	Variety (V)		RT×V	Rootstock (RT)	Variety (V)		RT×V
	= 0.04	0.05		0.08	= 0.12	0.15		0.21
	Shape index							
**Sukkary**	1.52	1.49	1.55	1.52A	1.49	1.49	1.53	1.50B
**Zebda**	1.51	1.55	1.52	1.52A	1.46	1.54	1.61	1.54A
**Mean**	1.51b	1.52ab	1.53a		1.47c	1.52b	1.57a	
**LSD at**_**0.05**_	Rootstock (RT)	Variety (V)		RT×V	Rootstock (RT)	Variety (V)		RT×V
	= 0.010	0.012		0.018	= 0.017	0.021		0.029

### Fruit chemical characteristics

Fruit chemical characteristics were significantly affected by rootstock, scion cultivar, and their interaction (Table 6). Irrespective of scion cultivar, the Sukkary rootstock produced the highest values of total soluble solids (TSS), titratable acidity (TA), TSS/TA ratio, total sugars, and reducing sugars, except in the 2024 season, where Sukkary and Zebda rootstocks showed statistically comparable values in TSS/TA ratio. With respect to scion cultivar effects, Osteen exhibited superior performance for TA, total sugars, and reducing sugars in both seasons, as well as higher TSS and TSS/TA ratio during the 2024 season. In contrast, R2E2 recorded the highest TSS values in 2025, while Tommy Atkins showed the greatest TSS/TA ratio in the same season. The Osteen/Sukkary combination recorded the highest levels of TSS, TA, total sugars, and reducing sugars. However, Osteen fruits grafted onto Zebda rootstock exhibited a higher TSS/TA ratio.

**Table 6:** Total soluble solids, titratable acidity, TSS/ titratable acidity ratio, total sugars, and reducing sugars of Tommy Atkins Osteen and R2E2 mango cultivars grafted on Sukkary and Zebda rootstocks during 2024 and 20252 seasons. Mean values representing different small letters, capital letters in columns and rows are significant between columns and rows, respectively, according to LSD (*p* < 0.05).

	**Season 2024**				**Season 2025**			
**Rootstock** **/** **Scion**	**Tomy Atkins**	**Osteen**	**R2E2**	**Mean**	**Tomy Atkins**	**Osteen**	**R2E2**	**Mean**
	Total soluble solids (%)							
**Sukkary**	16.83	18.38	15.73	16.98A	16.98	18.50	15.80	17.09A
**Zebda**	16.45	17.75	14.78	16.33B	16.65	17.05	14.78	16.16B
**Mean**	16.64b	18.06a	15.25c		16.81c	17.78b	15.29a	
**LSD at**_**0.05**_	Rootstock (RT)	Variety (V)		RT×V	Rootstock (RT)	Variety (V)		RT×V
	= 0.15	0.19		0.26	= 0.14	0.18		0.25
	Titratable acidity (%)							
**Sukkary**	0.29	0.34	0.28	0.30A	0.29	0.35	0.29	0.31A
**Zebda**	0.29	0.29	0.30	0.29B	0.29	0.28	0.31	0.29B
**Mean**	0.29b	0.31a	0.29b		0.293b	0.318a	0.295b	
**LSD at**_**0.05**_	Rootstock (RT)	Variety (V)		RT×V	Rootstock (RT)	Variety (V)		RT×V
	= 0.003	0.004		0.005	= 0.004	0.006		0.008
	TSS/titratable acidity ratio							
**Sukkary**	57.72	54.06	56.06	55.95A	59.15	52.49	55.45	55.69A
**Zebda**	57.42	62.23	48.85	56.17A	56.63	59.93	48.45	55.00B
**Mean**	57.57a	58.14a	52.46b		57.89a	56.21b	51.95c	
**LSD at**_**0.05**_	Rootstock (RT)	Variety (V)		RT×V	Rootstock (RT)	Variety (V)		RT×V
	= 0.68	0.83		1.18	= 0.53	0.65		0.92
	Total sugars (%)							
**Sukkary**	13.53	14.73	13.18	13.81A	13.60	14.40	13.30	13.77A
**Zebda**	13.43	13.73	13.08	13.41B	13.48	13.75	13.15	13.46B
**Mean**	13.48b	14.23a	13.13c		13.54b	14.08a	13.23c	
**LSD at**_**0.05**_	Rootstock (RT)	Variety (V)		RT×V	Rootstock (RT)	Variety (V)		RT×V
	= 0.13	0.16		0.23	= 0.12	0.14		0.20
	Reducing sugars (%)							
**Sukkary**	4.88	5.95	4.60	5.14A	5.08	5.45	4.30	4.94A
**Zebda**	4.40	4.95	4.05	4.47B	5.03	4.90	5.20	4.71B
**Mean**	4.64b	5.45a	4.33c		5.05b	5.18a	4.25c	
**LSD at**_**0.05**_	Rootstock (RT)	Variety (V)		RT×V	Rootstock (RT)	Variety (V)		RT×V
	= 0.07	0.09		0.12	= 0.08	0.10		0.14

### Antioxidant compounds

Fruit antioxidant compounds were significantly influenced by rootstock, scion cultivar, and their interaction (Table 7). Regarding rootstock effects, Sukkary recorded the highest contents of vitamin C and total carotenoids, whereas it exhibited the lowest total phenolic content in 2024 and showed no significant difference from Zebda rootstock in 2025. Among scion cultivars, Osteen consistently recorded the highest levels of antioxidant compounds across both seasons. However, in 2024, R2E2 showed comparable performance to Osteen in terms of total phenolic content. The interaction between rootstock and scion cultivar revealed marked differences in antioxidant compound accumulation. In Osteen, the highest vitamin C content was recorded when grafted onto Sukkary rootstock in 2024 and onto Zebda rootstock in 2025. Additionally, the Osteen/Sukkary combination produced the highest total carotenoid content, while the Osteen/Zebda combination consistently recorded the highest total phenolic content in both seasons.

**Table 7:** Influence of rootstock, scion, and their interaction on antioxidant compounds of Tommy Atkins Osteen, and R2E2 mango cultivars grafted on Sukkary and Zebda rootstocks during 2024 to 20252 seasons.

	**Season 2024**				**Season 2025**			
**Rootstock** **/** ** Scion**	**Tomy Atkins**	**Osteen**	**R2E2**	**Mean**	**Tomy Atkins**	**Osteen**	**R2E2**	**Mean**
	Vitamin C (mg/100 g fresh weight)							
**Sukkary**	31.95	34.08	28.45	31.49A	31.23	31.25	28.78	30.42A
**Zebda**	30.83	32.13	27.10	30.02B	30.78	32.00	27.70	30.16B
**Mean**	31.39b	33.10a	27.78c		31.00b	31.63a	28.24c	
**LSD at**_**0.05**_	Rootstock (RT)	Variety (V)		RT×V	Rootstock (RT)	Variety (V)		RT×V
	= 0.10	0.13		0.18	= 0.15	0.18		0.26
	Total carotenoid (mg/100 g fresh weight)							
**Sukkary**	4.51	5.91	4.40	4.94A	4.66	5.60	4.58	4.95A
**Zebda**	4.28	4.69	4.05	4.34B	4.24	4.75	4.24	4.41B
**Mean**	4.40b	5.30a	4.23c		4.45b	5.18a	4.41b	
**LSD at**_**0.05**_	Rootstock (RT)	Variety (V)		RT×V	Rootstock (RT)	Variety (V)		RT×V
	= 0.07	0.08		0.12	= 0.07	0.08		0.12
	Total Phenolic (%)							
**Sukkary**	0.07	0.08	0.07	0.07B	0.07	0.08	0.07	0.074A
**Zebda**	0.08	0.09	0.07	0.08A	0.08	0.09	0.08	0.083A
**Mean**	0.075b	0.085a	0.071a		0.075b	0.085a	0.076b	
**LSD at**_**0.05**_	Rootstock (RT)	Variety (V)		RT×V	Rootstock (RT)	Variety (V)		RT×V
	= 0.001	0.002		0.003	= 0.001	0.002		0.003

### Principal component analysis (PCA)

PCA clearly discriminated among mango cultivar rootstock combinations in both seasons and illustrated the relationships between vegetative growth, nutritional status, yield components, and fruit quality attributes (Figs. 1 and 2). In the first season, PC1 and PC2 explained 81.8% of the total variance (59.0% and 22.8%, respectively), while in the second season, they accounted for 81.8% (64.7% and 17.1%, respectively), indicating high reliability of the biplot interpretation across years. In both seasons, PC1 was primarily associated with vegetative vigor, mineral composition, and fruit physical traits. Positive PC1 values were strongly correlated with N, P, K, Ca, Mg, fruit weight, fruit length, pulp percentage, canopy volume, and tree height. Accordingly, R2E2/Zebda consistently occupied the extreme positive side of PC1 in both seasons and showed close association with canopy volume, tree height, shoot increment, and mineral nutrients, reflecting superior vegetative growth and nutrient status. R2E2/Sukkary also loaded positively on PC1, though with slightly lower magnitude, indicating enhanced fruit weight and nutritional attributes. Tommy Atkins/Zebda was positioned in the same quadrant, confirming its association with vegetative vigor and fruit size traits. Conversely, negative PC1 values in both seasons were associated with fruit number, peel percentage, yield efficiency, TSS/TA ratio, and seed percentage. Tommy Atkins/Sukkary and Osteen/Zebda were positioned toward this region, indicating a stronger association with yield-related traits and certain fruit composition parameters rather than vegetative vigor. PC2 mainly discriminated treatments based on biochemical quality attributes. Positive PC2 loadings were strongly related to TSS, total sugars, reducing sugars, total carotenoids, vitamin C, total phenols, and titratable acidity. In the first season, Osteen/Sukkary was clearly positioned in the upper quadrant close to these quality vectors, indicating enhanced internal fruit quality. A similar trend was observed in the second season, where Osteen/Sukkary maintained proximity to TSS, total sugars, and carotenoids, confirming the stability of its quality performance. In contrast, treatments positioned in the lower quadrant (*e.g.*, R2E2/Zebda in both seasons) were more closely associated with shoot increment and canopy expansion rather than biochemical enhancement.

**Figure 1:**
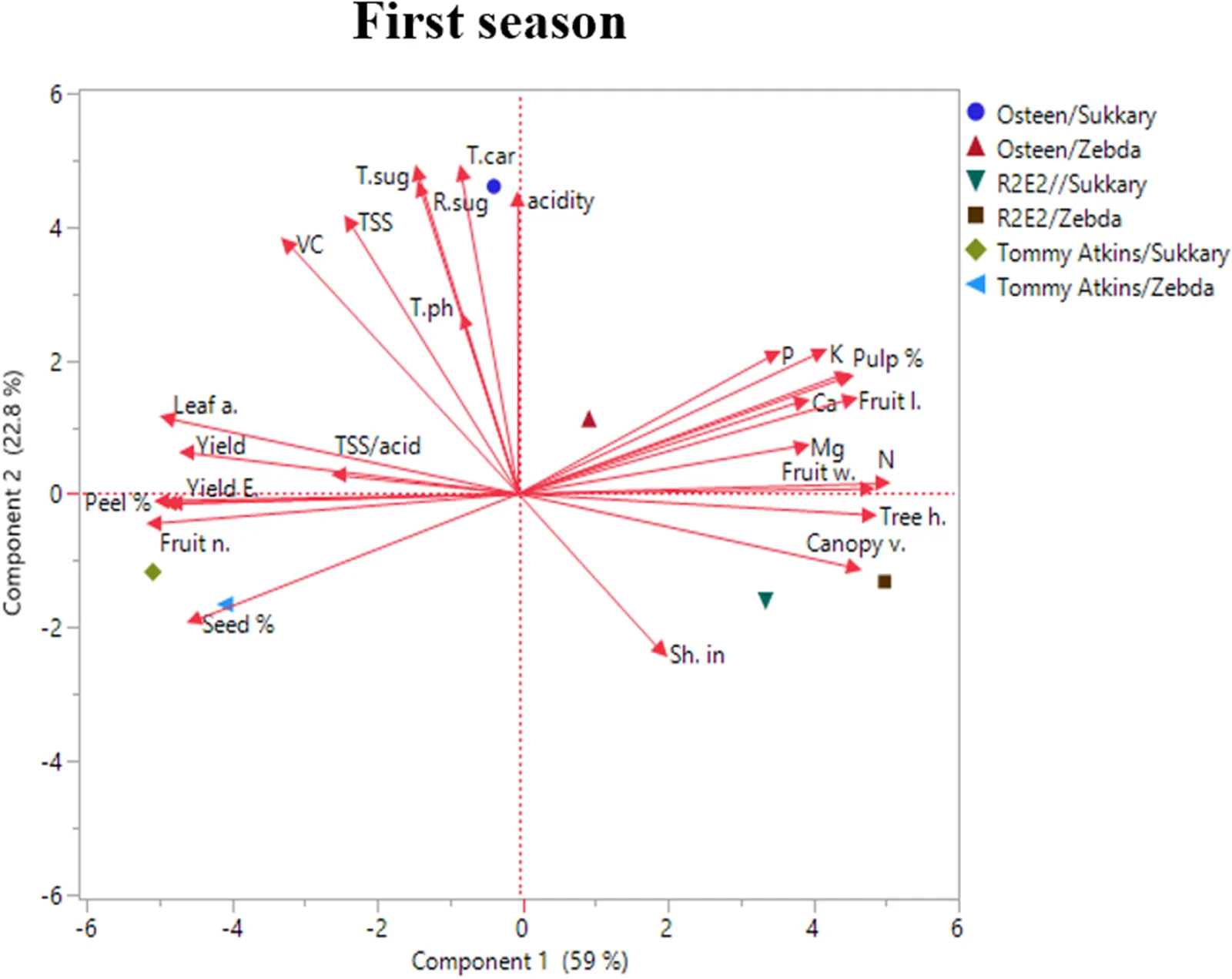
Biplots of principal component analysis (PCA), comparing the effects of rootstock, scion, and their interaction on various studied attributes of ‘Tommy Atkins’, ‘Osteen’ and ’R2E2’ mangoes grafted onto Sukkary and Zebda during the 2024 season. Tree height (Tree h.), Trunk diameter (Trunk d.), Canopy volume (Canopy v), Leaf area (leaf a.), Fruit number (Fruit n.), Yield efficiency (Yield E.), Fruit weight (Fruit w.), Fruit length (Fruit l.), Fruit width (F. w.), Shape index (Sh n.), Total sugars (T.sug), Reducing sugars (R.sug), Vitamin C (VC), Total carotenoid (T.car), Total Phenolic (T.p).

**Figure 2:**
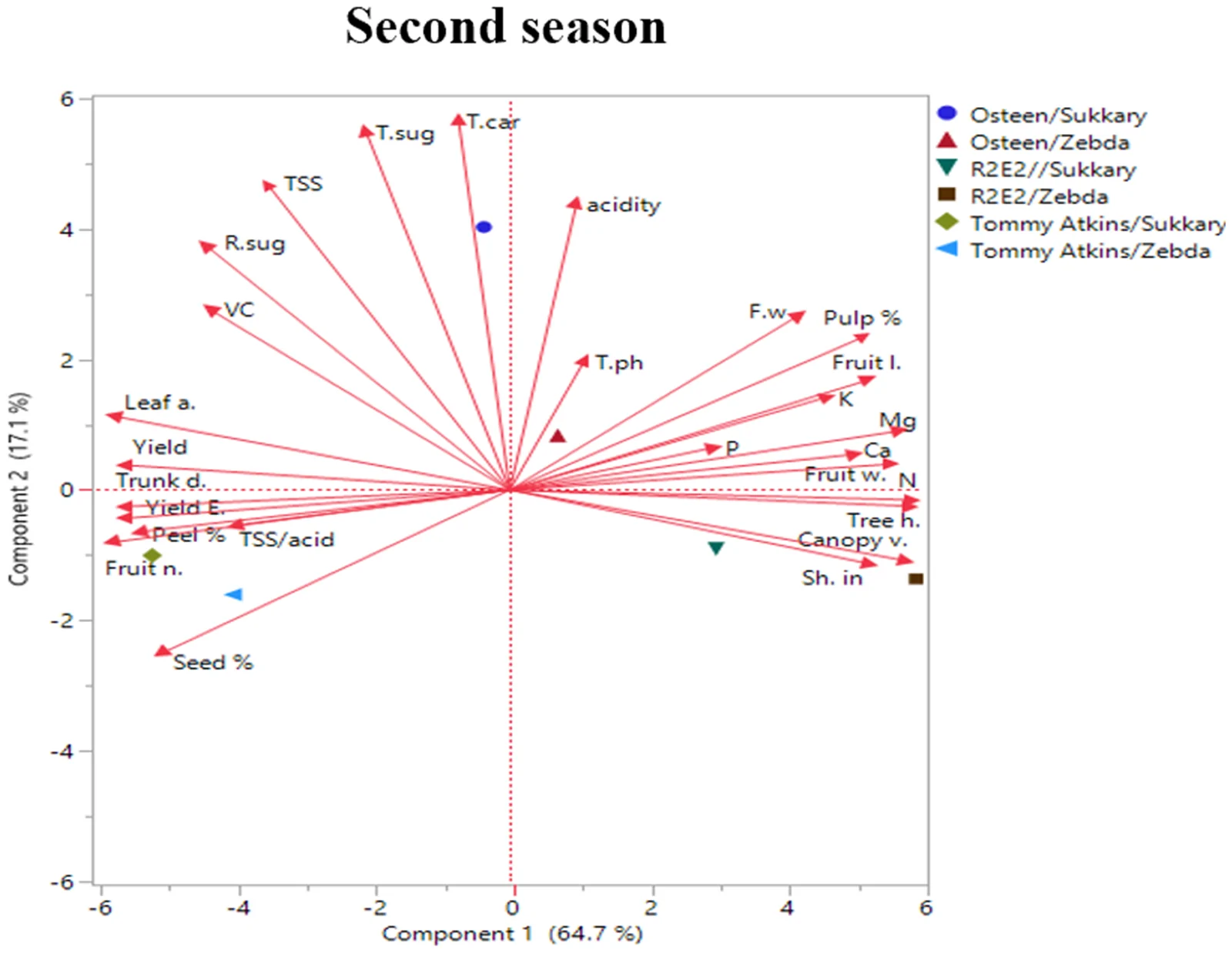
Biplots of principal component analysis (PCA), comparing the effects of rootstock, scion, and their interaction on various studied attributes of ‘Tommy Atkins’, ‘Osteen’ and ’R2E2’ mangoes grafted onto Sukkary and Zebda during the 2025 season. Tree height (Tree h.), Trunk diameter (Trunk d.), Canopy volume (Canopy v), Leaf area (leaf a.), Fruit number (Fruit n.), Yield efficiency (Yield E.), Fruit weight (Fruit w.), Fruit length (Fruit l.), Fruit width (F. w.), Shape index (Sh n.), Total sugars (T.sug), Reducing sugars (R.sug), Vitamin C (VC), Total carotenoid (T.car), Total Phenolic (T.p).

Overall, the PCA biplots demonstrated a consistent seasonal pattern: Zebda rootstock favored vegetative growth, mineral accumulation, and fruit size, particularly with R2E2, whereas Sukkary rootstock, especially when grafted with Osteen, was more closely associated with improved biochemical fruit quality. This clear separation across seasons confirms that rootstock–scion interaction plays a decisive role in modulating the balance between vegetative vigor, yield components, and internal fruit quality in mango.

## Discussion

### Tree growth

Both rootstock and scion varieties significantly influenced tree growth, likely due to differences in their genetic backgrounds. Enhanced growth in specific rootstock–scion combinations may be associated with increased chlorophyll content and higher photosynthetic rates, as reported previously (Rouphael, Kyriacou & Colla, 2018), although these parameters were not directly measured in the present study. In the present study, significant variations were observed in scion trunk diameter, tree height, and leaf area among the tested mango varieties as influenced by rootstock. Across two seasons, Tommy Atkins grafted onto Sukkary rootstock exhibited greater trunk diameter and leaf area. In contrast, R2E2 grafted onto either Sukkary or Zebda rootstocks demonstrated the greatest tree height and canopy volume. These findings suggest that vigorous rootstocks such as Sukkary and Zebda are well adapted to Tommy Atkins and R2E2 scions, indicating strong graft compatibility and efficient root system performance. Similar variations in mango growth parameters due to rootstock effects have been reported by Sharma, Dubey & Awasthi (2015). The enhanced growth observed in specific rootstock–scion combinations may be associated with increased chlorophyll content and higher photosynthetic rates, as photosynthetic efficiency is widely recognized as a key physiological indicator of plant vigor (Dayal et al., 2016) previously suggested that alterations in scion growth are largely dependent on the compatibility between rootstock and scion, as well as the structural and functional characteristics of the root system. Differential growth responses may also be influenced by the inherent growth habit of the scion cultivar and its capacity for endogenous hormone transport and long-distance signaling between roots and shoots. Hormonal communication plays a crucial role in coordinating root and shoot development. Earlier studies have identified auxin as a key signaling molecule transported from shoot to root *via* the phloem (Bithell et al., 2016; Tsaballa et al., 2021). Auxin levels in the roots regulate root growth and influence cytokinin synthesis and translocation (Shivran et al., 2023), thereby affecting overall tree vigor.

### Leaf tissue nutrient contents

Rootstocks significantly affected leaf nutrient composition across all scion varieties. In the present study, leaf concentrations of nitrogen (N), phosphorus (P), potassium (K), calcium (Ca), and magnesium (Mg) were consistently higher when Sukkary was used as the rootstock, regardless of the scion cultivar. Overall, the Zebda/R2E2 combination demonstrated strong nutrient-accumulation capacity, particularly for N, P, K, Ca, and Mg. The differential response of rootstocks in nutrient accumulation among scion varieties may be attributed to variations in vigor and root system characteristics. Both scion vigor and root system morphology play critical roles in nutrient uptake and translocation in grafted fruit trees (Kumar et al., 2017). Differences among rootstock genotypes may influence nutrient transport due to variation in xylem vessel dimensions (Dayal et al., 2017), as well as morphological and physiological root traits that directly affect ion absorption, redistribution, and translocation (Mestre et al., 2015), ultimately impacting plant growth (Nawaz et al., 2016). Differences in leaf nutrient concentrations among trees grafted on various rootstocks may indicate inherent variation in nutrient uptake efficiency. Alternatively, lower leaf nutrient concentrations in some combinations may result from a dilution effect associated with vigorous vegetative growth induced by certain rootstocks (Mestre et al., 2017).

### Yield component

Previous studies investigating rootstock effects on mango yield have reported contrasting findings. Some researchers indicated that yield is positively correlated with tree size, suggesting that larger trees produced by vigorous rootstock–scion combinations tend to achieve higher yields (Dubey et al., 2021; Adhikari, Xu & Notaguchi, 2022). Conversely, others have demonstrated that productive efficiency is not solely dependent on tree size, indicating that high yields can also be obtained from less vigorous but efficient graft combinations (Abewoy, 2021). In the present investigation, trees with larger trunk diameters on vigorous rootstocks provided greater bearing surface area and consequently produced more fruit. Moreover, cultivars grafted onto vigorous rootstocks exhibited greater leaf area, leading to increased photoassimilate production to support fruit development and ultimately enhance yield. These findings are consistent with the results reported by Dubey et al. (2021) and Hamed et al. (2021). Fruit yield per tree varied significantly depending on rootstock, scion variety, and their interaction. Regardless of rootstock, Sukkary proved to be the most productive cultivar, exhibiting the highest yield per tree, whereas Zebda recorded the lowest productivity. Furthermore, Tommy Atkins was identified as a highly prolific bearer, as trees of this cultivar produced the highest fruit yield per tree irrespective of rootstock. Considering rootstock effects on specific scion varieties, the Sukkary rootstock produced the highest yield per tree when grafted with Tommy Atkins. Variations in rootstock morphology, physiology, and growth characteristics likely contributed to differences in tree growth, graft compatibility, and fruiting potential, as previously noted by Rebolledo-Martínez et al. (2019). Differences in mango yield attributable to rootstock influence have also been reported by Galán Saúco (2018) and Vahdati et al. (2021). Variations in yield and yield efficiency may be attributed to differences in climatic conditions, as well as to modifications in tree morphology and rootstock physiology. These effects are reflected in tree vigor, scion–rootstock compatibility, and the fruiting potential of specific scion varieties grafted onto particular rootstocks (Tietel et al., 2020). The alkaline, moderately sodic soil conditions at the experimental site may have influenced nutrient availability, particularly for P and K, and should be considered when interpreting rootstock performance.

### Fruit physical characteristics

The individual effects of rootstock and scion variety significantly influenced most of the evaluated fruit quality attributes. Sukkary rootstock consistently enhanced fruit weight, pulp weight, and fruit dimensions irrespective of the scion cultivar. However, the interaction between rootstock and scion exhibited variable and sometimes inconsistent effects on certain fruit quality parameters. In the cultivar R2E2, fruits produced on Sukkary rootstock showed higher fruit weight, pulp weight, and fruit dimensions, whereas peel content and seed weight were not significantly affected. In Osteen, the influence of the Zebda rootstock was observed only for the shape index during the first season. Previous studies have similarly reported variable rootstock effects on mango fruit quality: for example, Paolo Mauro et al. (2020) observed no significant effect of rootstock on fruit weight in Alphonso, whereas De Carvalhoa et al. (2019) reported significant rootstock effects on fruit size and weight in a sweet orange cultivar.

### Fruit chemical characteristics

In the present study, most fruit chemical characteristics were significantly influenced by rootstock in both seasons. Sukkary rootstock produced fruits with higher total soluble solids (TSS), TA, total sugars, and reducing sugars. However, the TSS/TA ratio was not significantly affected by rootstock during the first season. In the cultivar Osteen, fruits grafted onto Sukkary rootstock exhibited higher titratable acidity, TSS, total sugars, and reducing sugars. Seasonal variation also played a role in fruit quality attributes. In addition to genetic factors, environmental conditions are known to influence phytochemical composition substantially (Ogwu, Izah & Joshua, 2025). Dubey et al. (2021) emphasized that genotype is a major determinant of phytochemical content, although growing conditions can significantly modify these attributes. Similarly, Zhang et al. (2018) reported positive correlations between fruit quality and environmental factors such as sunshine duration and temperature during the growing season. In contrast, relative humidity showed a negative correlation with fruit quality parameters.

### Antioxidant compound

Our results indicated that vitamin C and total carotenoid contents were significantly affected by both rootstock and scion variety. Mango fruits grafted onto Sukkary rootstock exhibited higher vitamin C and total carotenoid concentrations compared to other rootstocks. In particular, the Sukkary rootstock proved superior for the cultivar Osteen, resulting in the highest vitamin C and total carotenoid content. These findings are consistent with previous reports by Hamed et al. (2021) and Shivran et al. (2022) regarding variations in vitamin C content, as well as Xiao & Hongwu (2013), who documented increased total carotenoid levels associated with specific rootstocks. Regarding total phenolic content (Table 7), the lowest phenol concentrations were recorded in fruits from trees grafted onto the vigorous Sukkary rootstock. In contrast, the highest total phenol content was observed in fruits grafted onto Zebda rootstock. These findings suggest a negative relationship between phenolic content and tree vigor. Similar conclusions were reported by Zayan et al. (2011) and Akin-Idowu et al. (2020), who identified associations between tree vigor and total phenolic content in mango. Murti & Upreti (2003) also reported comparable results across seven polyembryonic mango cultivars, which are commonly used as rootstocks. Differences in phenolic content attributable to rootstock effects have likewise been documented in sweet cherry (Gonçalves et al., 2005) and mango (Dubey et al., 2021). However, Kviklys et al. (2014) noted that the accumulation of certain compounds may depend primarily on rootstock genotype rather than on rootstock-induced scion vigor, indicating that biochemical responses may not always directly correspond to vegetative growth patterns.

## Conclusions

The present study demonstrates that mango rootstocks significantly influence scion vigor and fruit yield, while causing comparatively minor variations in fruit quality attributes. Rootstocks generally exhibited a strong capacity for absorbing nutrients, including nitrogen (N), phosphorus (P), potassium (K), calcium (Ca), and magnesium (Mg). Moreover, the findings indicate that nutrient uptake efficiency is not solely dependent on the root system; scion genotype also contributes to nutrient absorption and overall tree performance. After eight years of planting, Tommy Atkins grafted onto Sukkary rootstock exhibited a reduced canopy volume and superior fruit production. This combination, therefore, offers a practical advantage for orchard management, as it allows increased planting density without compromising fruit quality. Accordingly, grafting Tommy Atkins onto Sukkary rootstock is recommended to optimize productivity and improve planting efficiency under similar growing conditions. Nevertheless, further research is recommended to address the photosynthetic rate, stomatal conductance, and hormone concentrations that may explain the nutritional status, yield components, quality, and antioxidant performance.

## Supplemental Information

10.7717/peerj.21896/supp-1 Supplemental Information 1 Raw data

10.7717/peerj.21896/supp-2 Supplemental Information 2 Statistical analysis for the first year

10.7717/peerj.21896/supp-3 Supplemental Information 3 Statistical analysis for the second year

## Additional Information and Declarations

### Data Availability

The following information was supplied regarding data availability: The raw data are available in the Supplemental Files.
